# Mitochondrial genome and functional defects in osteosarcoma are associated with their aggressive phenotype

**DOI:** 10.1371/journal.pone.0209489

**Published:** 2018-12-21

**Authors:** Martina Jackson, Nicole Serada, Maura Sheehan, Satish Srinivasan, Nicola Mason, Manti Guha, Narayan Avadhani

**Affiliations:** Department of Biomedical Sciences, School of Veterinary Medicine, University of Pennsylvania, Philadelphia, Pennsylvania, United States of America; University of Texas Health Science Center at San Antonio, UNITED STATES

## Abstract

Osteosarcoma (OSA) is an aggressive mesenchymal tumor of the bone that affects children and occurs spontaneously in dogs. Human and canine OSA share similar clinical, biological and genetic features, which make dogs an excellent comparative model to investigate the etiology and pathogenesis of OSA. Mitochondrial (mt) defects have been reported in many different cancers including OSA, although it is not known whether these defects contribute to OSA progression and metastasis. Taking a comparative approach using canine OSA cell lines and tumor tissues we investigated the effects of mtDNA content and dysfunction on OSA biology. OSA tumor tissues had low mtDNA contents compared to the matched non-tumor tissues. We observed mitochondrial heterogeneity among the OSA cell lines and the most invasive cells expressing increased levels of OSA metastasis genes contained the highest amount of mitochondrial defects (reduced mtDNA copies, mt respiration, and expression of electron transport chain proteins). While mitochondria maintain a filamentous network in healthy cells, the mitochondrial morphology in OSA cells were mostly “donut shaped”, typical of “stressed” mitochondria. Moreover the expression levels of mitochondrial retrograde signaling proteins Akt1, IGF1R, hnRNPA2 and NFkB correlated with the invasiveness of the OSA cells. Furthermore, we demonstrate the causal role of mitochondrial defects in inducing the invasive phenotype by Ethidium Bromide induced-mtDNA depletion in OSA cells. Our data suggest that defects in mitochondrial genome and function are prevalent in OSA and that lower mtDNA content is associated with higher tumor cell invasiveness. We propose that mt defects in OSA might serve as a prognostic biomarker and a target for therapeutic intervention in OSA patients.

## Introduction

Osteosarcoma (OSA) is an aggressive neoplasia of the bone, which affects human children and older, large and giant breed canines [1–3]. It accounts for about 85% of all primary bone tumors in both species [4]. The malignant neoplasm arises from osteoblasts and can manifest as both osteoproductive and osteolytic lesions [4]. In both species, the tumor most commonly occurs in the metaphyseal regions of the long bones including the humerus, femur, radius, tibia, and ulna [5–7]. In human patients, treatment consists of neo-adjuvant chemotherapy followed by radical surgery. In canines current OSA treatment involves limb amputation, chemotherapy, and palliative radiation [8–12]. Even with aggressive treatment strategies, metastatic disease leads to high mortality rates in humans and canines [13–15]. The poor prognosis makes it imperative to investigate the etiology and pathogenesis of OSA to identify new molecular markers and design effective treatments for both human and canine patients. Given the similarities in the occurrence, biology, behavior and molecular features between human and canine OSA, identification of novel prognostic markers and therapeutic targets explored in either species can be evaluated further for their relevance in the comparative model for developing treatment modalities.

Mitochondrial dysfunction caused by mtDNA mutations, deletions, and depletion have been widely reported in different types of cancers including OSA [16,17]. It is reported that tumors with an aggressive phenotype have impaired mitochondrial function and increased glycolytic metabolism [18–28]. Reports suggest that mtDNA mutations and electron transport chain (ETC) complex defects can enhance tumor aggressiveness through increased ROS production, constitutive activation of nuclear genes involved in cell survival and angiogenesis, and resistance to apoptosis [29,30]. We earlier reported that mitochondrial dysfunction, and the subsequent loss of transmembrane potential (ΔΨm), elevates cytosolic Ca^2+^ levels and activates the calcineurin dependent mitochondria-to-nucleus retrograde signaling (MtRS) pathway [31,32]. This pathway activates the nuclear transcription factors NFκB, C/EBPδ, CREB, and NFAT resulting in transcriptional activation of oncogenes such as Akt1, glucose transporter (Glut4), Ryanodine receptor (RyR1) and Cathepsin L [33–35]. Furthermore, hnRNPA2, an RNA-binding protein, is activated in response to mtDNA depletion and acts as a transcriptional coactivator to propagate MtRS [34–36]. Indeed, consistent with these findings we have also found that reducing mtDNA copy number in different non-tumorigenic immortalized cell lines causes a metabolic shift to glycolysis, and results in their transformation to tumorigenic cells [20,31,32,34–37].

In many cancers, it has been reported that patients that have tumors with mtDNA defects have a poor prognosis [38–42]. We recently reported that aggressive breast tumors have higher prevalence of mitochondrial defects [27]. Here we investigated the contribution of mitochondrial dysfunction to the aggressive behavior of OSA with the goal of determining possible prognostic significance and identifying new druggable molecular targets for relapsed, aggressive OSA. We hypothesize that mtDNA defects contribute to OSA progression and metastasis and as such may represent a valuable prognostic biomarker and a potential therapeutic target for future exploration.

## Materials and methods

### Cell culture

The canine OSA cell lines SK-KOSA, MC-KOSA, and BW-KOSA were obtained from the NCI- Canine Comparative Oncolgy Genomics Consortium (CCOGC) [43]. Cells were cultured in Dulbecco Modified Eagle Medium (DMEM) with 10% fetal bovine serum (FBS), 1% Penicillin-Streptomycin, and 0.1% Fungizone. Reduction in mtDNA copy number was achieved by treating cell lines with 50 ng/mL of ethidium bromide (EtBr) for three passages. MtDNA depleted cells were supplemented with 1 mm pyruvate and 50 μg/ml uridine. Cells were stored in 37°C and 5% CO_2_ and were passaged at 70–80% confluence. MtDNA depleted cell lines were designated SK-KOSA+EB, MC-KOSA+EB, and BW-KOSA+EB.

### Canine OSA tissues

Canine primary tumor and non-tumor “control” tissues were obtained from canine OSA patients directly following limb amputation surgery. This research was approved by the University of Pennsylvania’s Institutional Animal Care and Use Committee (IACUC Protocol number: 806287). Biopsies were obtained from three different regions of the tumor to assess intra-tumor heterogeneity in mtDNA contents. For comparison of mtDNA content, non-tumor skeletal muscle tissue from two regions surrounding the tumor was used. Of the five patients that were included in this study, three had received only chemotherapy treatment with carboplatin at the time of tissue collection and two had received an experimental vaccine aimed at preventing metastatic disease, known as the listeria vaccine. Two patients later also received the vaccine. At the time this paper was written, all patients were alive with no evidence of metastatic disease.

### Relative mtDNA content

At least three punch sections were taken from each tissue for replicates to rule out the effects of intratumor heterogeneity and each sample was analyzed in triplicate. MtDNA copy number was measured from total genomic DNA isolated from normal and tumor tissues using DNeasy Blood and Tissue kit (Qiagen Cat # 69504). Using SYBR Green assay and quantitative real time PCR (qPCR) we amplified mtDNA (50 ng genomic DNA template) using primers specific for mtDNA encoded gene Cytochrome Oxidase subunit I (*MT-COI*) and normalized using a nuclear encoded single copy gene Cytochrome c Oxidase subunit IVi1 (*CcO IVi1*). Data analysis was done using the ΔΔCT method. This method of analysis estimates mtDNA copy number relative to a single copy nuclear gene as an internal reference gene [28,36,44]. Taking into consideration that tumors often have nuclear genome polyploidy due to chromosomal instability, in preliminary experiments, we used two nuclear encoded single copy genes, *36B4* and *CcOIVi1*, coded on different chromosomes, as reference genes for normalization. Although there are possible inter individual differences in the transcripts of nuclear coded genes in tumors, the overall patterns of mtDNA content among our comparisons remained unchanged irrespective of the gene of reference.

### Western immunoblot

Total cell lysates were prepared by solubilizing cell pellets in NP-40 lysis buffer (150mM NaCl, 1.0% NP-40, and 50mM Tris-Cl pH 8.0, containing protease inhibitors). 50μg protein were suspended in Laemmli sample buffer and resolved by electrophoresis on 12% SDS-polyacrylamide gels [45] and subjected to immunoblot analysis. The protein levels were estimated by the Lowry method [46]. The immunoreactivity of the primary antibodies used in this study had not been previously tested in canine samples, so the time and concentration of each antibody was standardized in preliminary experiments in canine lysates. All secondary antibodies were IgG antibodies from LI-COR Biosciences and were specific to the species of the primary antibody. The blots were analyzed on a LI-COR Odyssey Infrared Imaging System and quantitated using Image J. To confirm equal protein loading, blots were re-probed with actin antibody. The fluorescence intensity of each immunoreactive protein band was normalized to the fluorescence of the corresponding actin band. pAkt was first compared with actin, then this value was divided by Akt to show the comparative activation. SK- or BW-KOSA (as indicated in the figures) was set to one as a comparison. Representative blots from 3–4 independent experiments are presented here.

### Antibodies

All antibodies were diluted in 1x PBS+ 0.05% Tween20 (0.05%PBST).

hnRNPA2 (1:750): Santa Cruz Biotechnology, Dallas, TX; Cat # sc-32316.

IGF-1Rβ (1:2000): Santa Cruz Biotechnology, Dallas, TX; Cat # sc-713

Akt (1:2000): Santa Cruz Biotechnology, Dallas, TX; Cat # sc-1619

p-Akt (1:2000): Cell Signaling Technology, Danvers, MA; cat # 9271

p50 (1:1000): Santa Cruz Biotechnology, Dallas, TX; Cat # sc-8414

OPA1 (1:1000): Abcam, Cambridge, MA; Cat # ab157457

DRP1 (1:1000): Abcam, Cambridge, MA; Cat # ab184247

### Immunocytochemistry

Cells (2x10^5^ cells per well on a 6-well plate) were grown overnight in growth medium on Poly-D Lysine coverslips. Cell adherence and confluence was confirmed before processing. After 24h of cell seeding, cells were washed with 1X PBS and fixed in ice-cold methanol for 10 minutes, at room temperature. Fixed cells were blocked in a buffer containing 1% BSA and were incubated with primary antibodies (as indicated in the figures) for 1 hour at 37°C. Immunostained cells were imaged using a Leica confocal microscope under a 100X objective.

### Mitochondrial respiration

Oxygen consumption rate (OCR) and extracellular acidification rate (ECAR) were carried out in a XF24 Seahorse Analyzer (Seahorse Bioscience, Billerica, MA, USA) using 2.5 × 10^3^ cells as described before [20,27,28]. An equal number of cells was counted and seeded on the XF24 culture plates 6h prior to performing the assay to rule out differences in proliferation rates between cell types. Thirty minutes prior to the OCR and ECAR measurements, the growth medium of the cells was replaced with unbuffered assay medium containing 10 mM glucose in the absence of CO_2_.

### Growth curve

Growth patterns were measured by seeding cells at a density of 1 × 10^4^ cells/well in 24-well culture plates as described earlier [34]. Cells from three wells at each time point were harvested and counted in a Guava personal cytometer according to the manufacturer’s protocols (Millipore, Billerica MA).

### Matrigel invasion assay

The *in vitro* invasion assays were carried out as described previously [47]. The Matrigel invasion chambers were prepared at 1:2 dilution of Matrigel (BD Biosciences, Belford, MA) as described before [34]. Equal numbers of viable cells (4 × 10^4^) were seeded on top of the Matrigel layer. After incubation for 24 h at 37°C, non-invading cells in the Matrigel layer were quantitatively removed, and the microporous membrane containing cells that migrated across the Matrigel layer and through the microporous membrane was stained and viewed under an Olympus BX 61 bright field microscope as described before [34]. At least six fields were examined within any one experiment for each condition and a representative image for each cell line is presented.

### Quantitative real time PCR

Total cellular RNA was prepared using the RNeasy mini kit (Qiagen Cat # 74104). Genomic DNA was eliminated from the RNA preparations using Turbo DNA Free kit (Thermo Fisher Scientific). 1μg RNA was reverse transcribed into cDNA using High Capacity reverse transcription kit (Applied Biosystems). 100 ng cDNA was used for each SYBR Green reaction for transcript analysis of Ezrin, β4 Integrin and Cullin-1. Quantitative Real Time PCR assays were run on an ABI Quant Studio 6 real time thermocycler (Applied Biosystems). The nuclear gene GAPDH was used as an endogenous control. All real time PCR assays were run in triplicate. Data are presented as Relative Quantification (RQ).

The primer sequences used are as follows:

### Ezrin

Forward primer 5’-GTTGATGCCCTTGGAC-3’,

Reverse primer 5’-GGTGCCTTCTTGTCG-3’

### β4 Integrin

Forward primer 5’-TCATGAGCCGCAATGATGAG-3’,

Reverse primer 5’-TGATGGATAGTCCTGTGTCTTGTACTG-3’

### Cullin-1

Forward primer 5’-CTTAGAAGCCCAGTCAACACCAT-3’,

Reverse primer 5’-GGCCTTTCACAATATTTGCCATA-3’

### Statistical analysis

Statistical analysis was done using one-tail paired Student’s t-test. P value of <0.05 was considered as statistically significant and indicated by an asterisk (*).

## Results

### Inherent heterogeneity in mtDNA content among osteosarcomas

**Fig 1:**
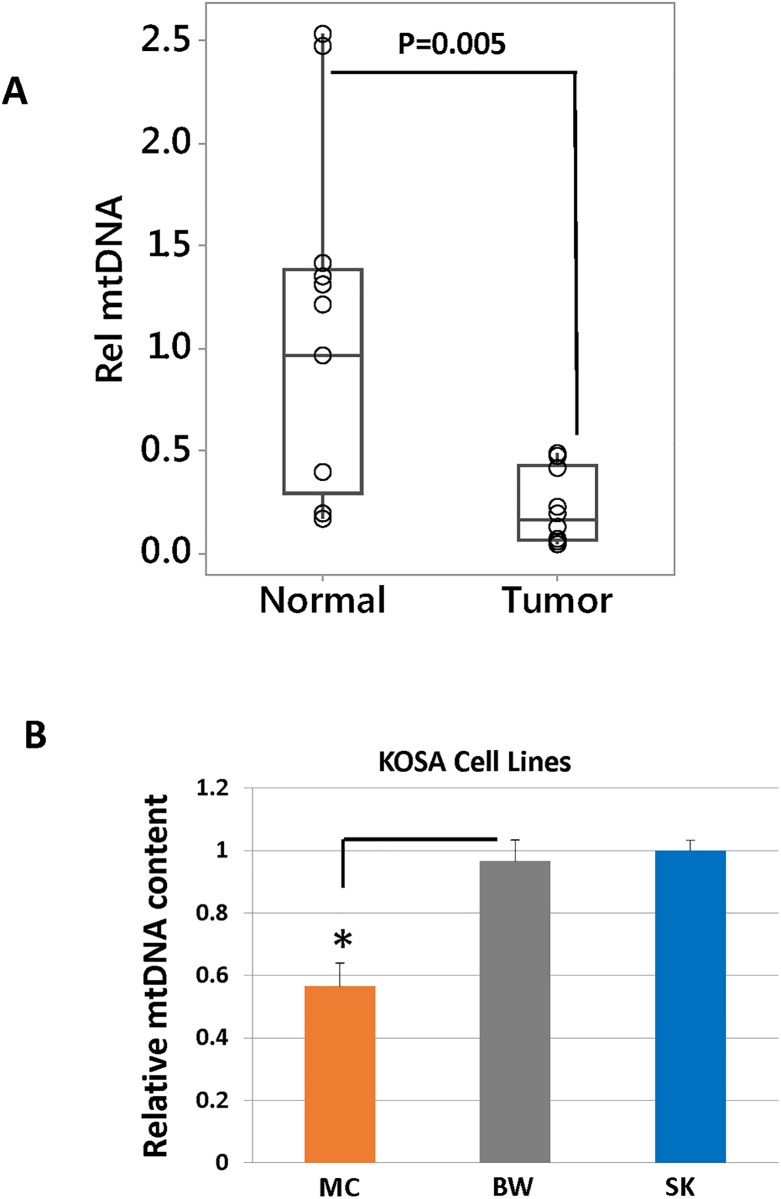
Differences in mtDNA content in OSA cell lines and tissue samples. Real time PCR showing relative mtDNA levels in **(A)** OSA tumor tissues (n = 10) and matched non-tumor tissues (n = 10), **(B)** MtDNA estimation in OSA cell lines using real time PCR. The mtDNA content (y-axis) is quantified from total DNA as the copy number of mtDNA gene CcO1, normalized to the copy number of nuclear single copy gene CcOIVi1.

We analyzed five canine patient OSA tumor tissues for their mtDNA content by comparing mtDNA encoded CcO1 gene to the nuclear encoded CcOIVi1 gene. To exclude the possibility that intra-tumor heterogeneity can account for the variations in mtDNA content, we have analyzed atleast 3 punch biopsies from each tumor and non-tumor tissue. The mtDNA copy numbers between the different punch biopsies from the same patient remained similar with no statistical difference. Non-tumor muscle tissue (of mesenchymal origin, as is osteosarcoma) from the same patient was used as a control. We observed that OSA tumors had lower mtDNA content than non-tumor tissues taken from the same patient (Fig 1A). There is heterogeneity in the mtDNA content among both tumor as well as non-tumor “control” samples. We used statistical analysis to conclude that the mtDNA content is lower in tumor tissues compared to the non-tumor normal tissues.

We next analyzed three canine OSA cell lines and observed heterogeneity in mtDNA copy numbers among these cells. Comparisons between the ‘parental’ cell lines were made to determine inherent mitochondrial heterogeneity among different OSA cells. MC-KOSA inherently had lower mtDNA content than SK-KOSA and BW-KOSA cells (Fig 1B). This suggests that there is inherent variation in mtDNA levels among osteosarcomas which could potentially determine OSA aggressiveness.

### Reduced mtDNA content correlates with altered mitochondrial morphology, dynamics and respiratory function in OSA cells

To investigate the causal role of mtDNA defects, we partially depleted mtDNA copy number (90% depleted compared to the parental mtDNA content) in the OSA cell lines (SK-KOSA, MC-KOSA, and BW-KOSA) by ethidium bromide (EtBr) treatment as previously described [28,31,32]. As reported earlier, these treatments do not affect nDNA content [28,48–51]. The resulting SK-KOSA+EB, MC-KOSA+EB, and BW-KOSA+EB cell lines each contained 10% of their parental mtDNA content (S1 Fig).

**Fig 2:**
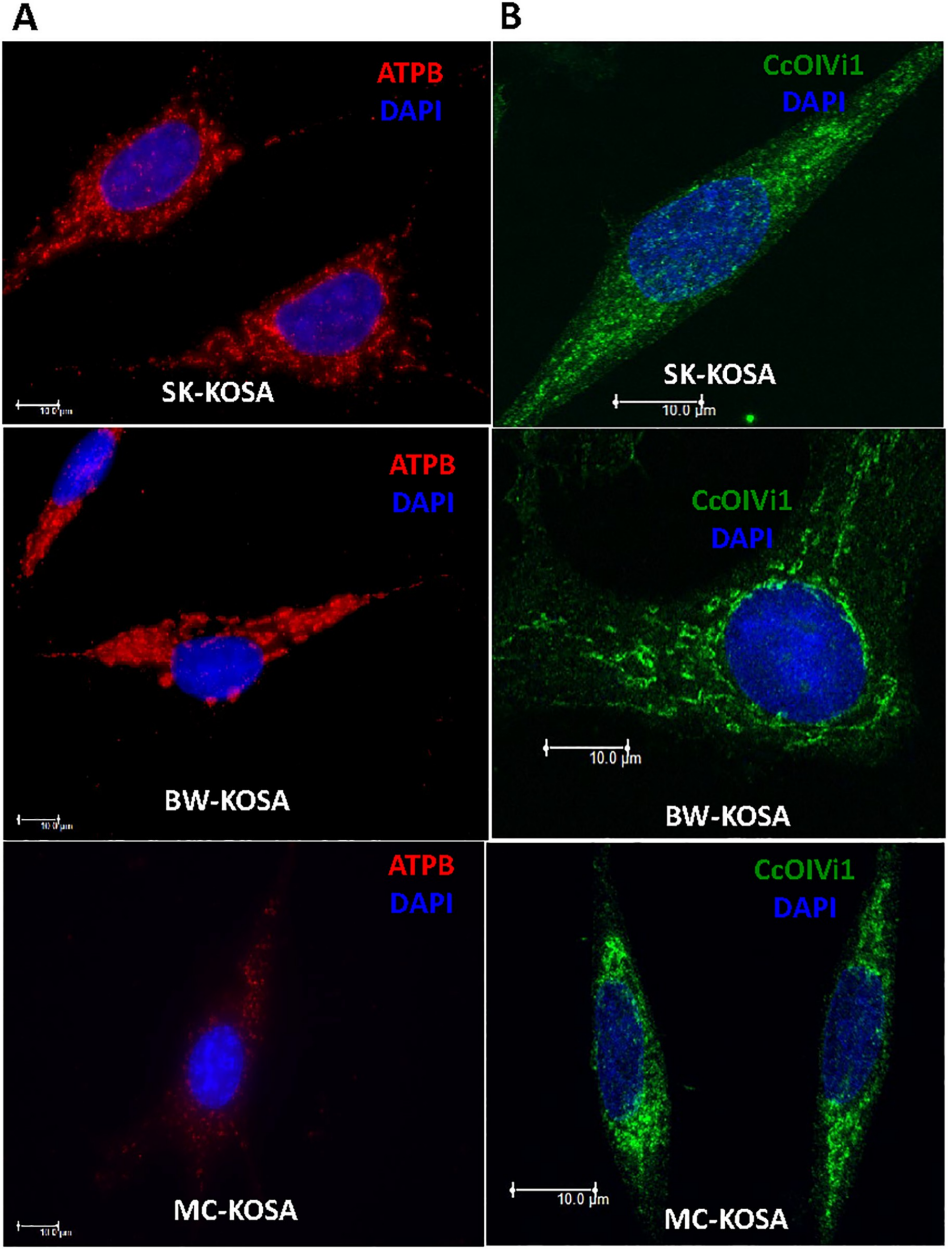
Expression of electron transport chain proteins in OSA cell lines. **(A)** Immunofluorescence images showing ATPB (an electron transport chain protein in red) and nuclei (DAPI, blue) staining pattern in parental OSA cell lines. **(B)** OSA cell lines stained for CcOIVi1 (green) and nuclei (DAPI, blue). Scale bar: 10μm, magnification 100x.

We hypothesized that aggressive OSA tumors have mitochondrial defects that potentially contribute to their aggressiveness. We investigated whether there is a correlation between mtDNA content and mitochondrial morphological alterations (fragmentation, loss of filamentous network) in the different OSA cell lines. Healthy functional mitochondria have filamentous network morphology and under cellular stress conditions such as hypoxia or osmotic pressure changes, mitochondria acquire a “donut-shaped” morphology [52,53]. Mitochondrial fragmentation has been reported to occur under different physiological or pathological conditions. We observed the presence of “donut-shaped” circular mitochondria along with filamentous mitochondria in the three OSA cell lines and MC-KOSA cell line which had the lowest mtDNA content showed the highest prevalence of circular “donut-shaped” mitochondria and a loss of mitochondrial network. We also found that expression of the electron transport chain (ETC) proteins ATP5B (Fig 2A) and CcOIVi1 (Fig 2B) was reduced in MC-KOSA cells compared to SK-KOSA and BW-KOSA cells.

Furthermore, experimentally reducing mtDNA content, by EtBr treatment, in these cell lines resulted in loss of mitochondrial filaments suggesting that reduction in mtDNA content impacts mitochondrial morphology. The expression of mitochondrial proteins ATP5B and CcOIVi1 is markedly reduced in the mtDNA-depleted cell lines compared to the corresponding parental cells (S2 Fig). This suggests that reduced mtDNA content correlates with and plays a causal role in the loss of mitochondrial filaments and reduced levels of electron transport chain proteins which are essential to maintain normal mitochondrial function.

**Fig 3:**
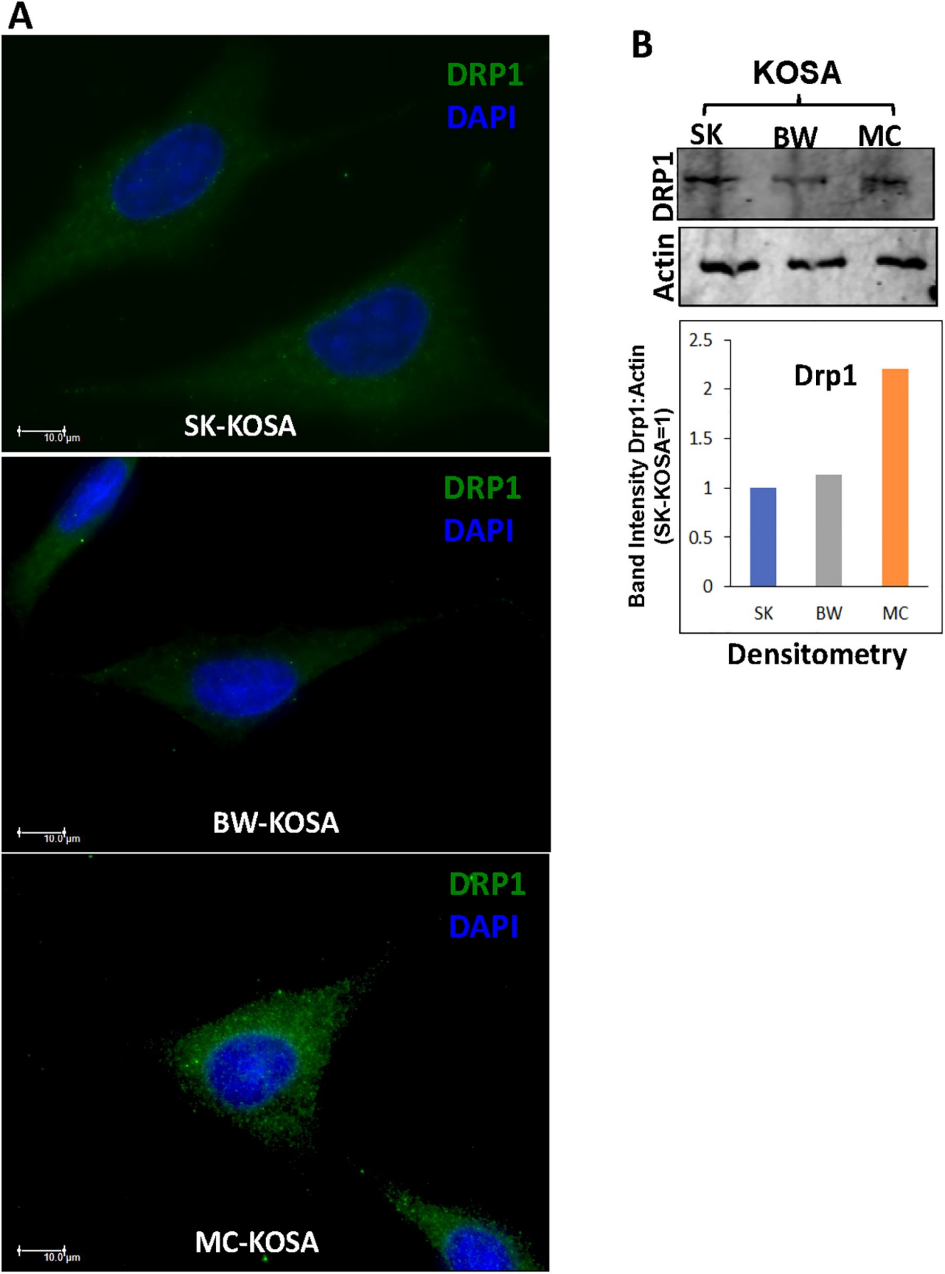
Altered expression of mitochondrial fission marker in OSA cell lines. **(A)** Immunocytochemistry of OSA cell lines stained for mitochondrial fission marker protein Drp1 (green) and nuclei (DAPI, blue) viewed under a Leica widefield microscope. Scale bar: 10μm, magnification 100x. **(B) Top panel**: Western Immunoblot showing high DRP1 protein levels in MC-KOSA relative to SK-KOSA and BW-KOSA. **Bottom Panel**: Quantitation (densitometry) of the protein levels from the western immunoblot.

Mitochondria are highly dynamic cell organelles, and a balance of mitochondrial fusion and fission is critical for maintaining mitochondrial functions and cellular signaling [54–57]. Several proteins have been reported to be involved in mitochondrial fusion-fission dynamics, including OPA1, which controls inner membrane fusion, and Drp1, which is recruited to the outer membrane for fission [21,58–61]. By immunocytochemistry as well as immunoblot, we observed elevated levels of mitochondrial fission marker DRP1 (Fig 3A and 3B) and lower level of mitochondrial fusion marker OPA1 (S3 Fig) in MC-KOSA cells compared to SK-KOSA and BW-KOSA cells. This suggests that lower mtDNA content is associated with higher mitochondrial fission in MC-KOSA cells and that reduction in mtDNA content influences mitochondrial dynamics.

**Fig 4:**
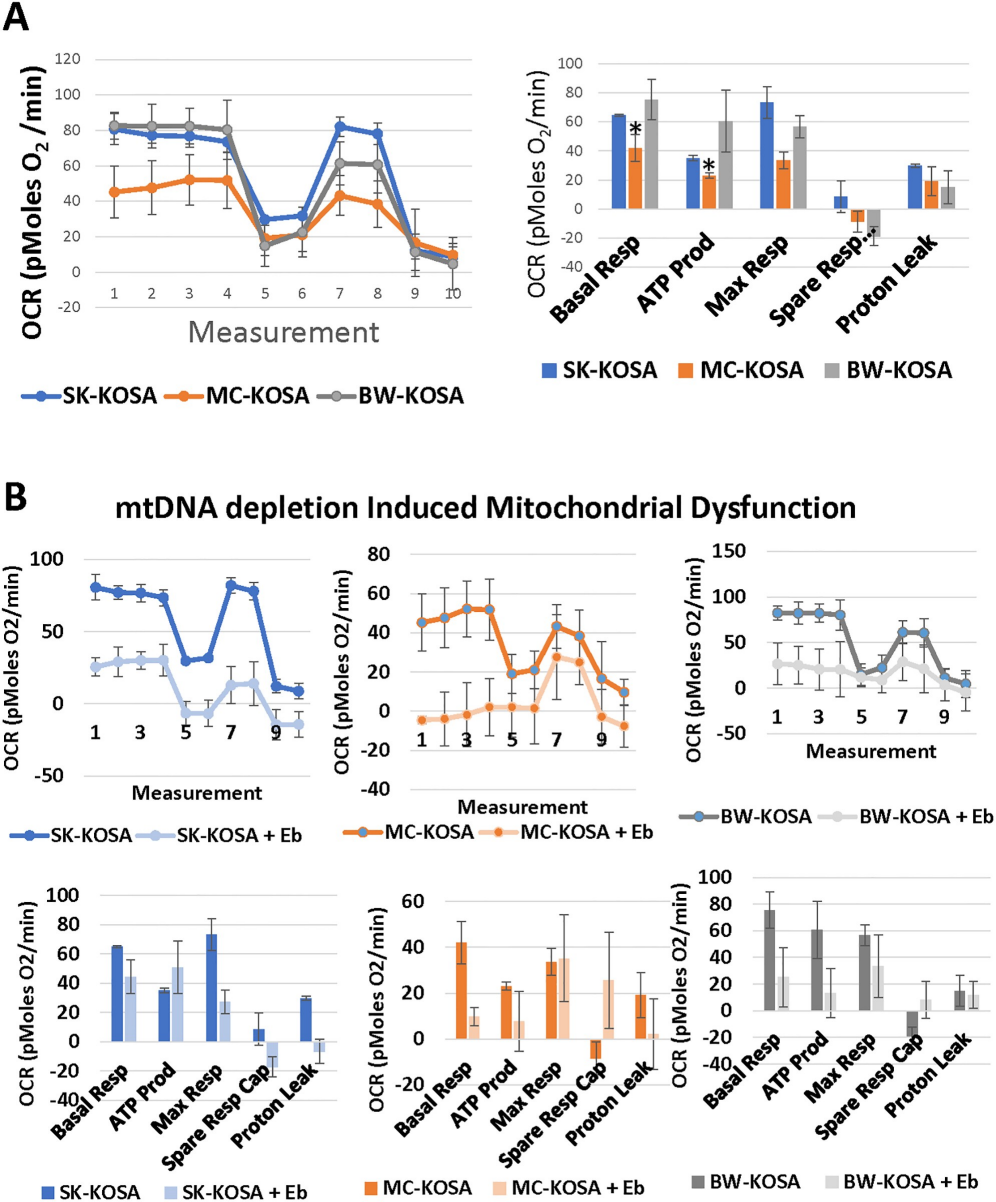
Heterogeneity in cellular respiratory capacity in parental and mtDNA-depleted OSA cell lines. **(A)** Seahorse XF24 was used to analyze differences in respiration parameters among parental OSA cell lines and **(B)** among parental OSA cell lines compared to mtDNA-depleted cell lines. Oligomycin (2 μg/mL) was added at step 4, FCCP (0.3 μM) at step 6, and Rotenone (1 μM) at step 8.

To evaluate the mitochondrial functions, we analyzed the cellular respiratory capacity of KOSA cell lines using a Seahorse Flux Analyzer. The MC-KOSA cell line which contained the lowest mtDNA content and most dramatic loss of the mitochondrial network, had the lowest mitochondrial respiratory capacity (Fig 4A). Reduction of mtDNA content further impaired mitochondrial respiratory function in SK-KOSA-EB, MC-KOSA-EB, and BW-KOSA-EB respectively (Fig 4B). Additionally, EtBr mediated mtDNA depletion further reduced mitochondrial respiration and also reduced basal respiration, ATP production, and maximal respiration in SK-KOSA-EB, MC-KOSA-EB, and BW-KOSA-EB compared to their parental cell lines (Fig 4A–4B). These results suggest that mitochondrial functional defects observed in OSA are associated with low mtDNA contents, and that the degree of functional impairment correlates with the loss of mtDNA copies.

### Mitochondrial defects correlate with the activation of nuclear protein markers of mitochondrial retrograde signal

We previously reported in multiple immortalized murine and human cells and cancer cell lines that calcium-calcineurin mediated retrograde signaling (MtRS) is activated in response to mtDNA depletion, loss of electron transport chain proteins and disruption of mitochondrial membrane potential [21,31,32,34,37,62]. We have shown that dysfunctional mitochondria induce a mitochondria-to-nucleus retrograde signaling (MtRS) pathway that results in the activation of PI3-kinase, IGF-1R, and Akt1, which are all involved in metabolic shift to preferentially glycolysis and tumorigenic phenotype [19,34,35,37]. Nuclear transcription factor NFkB (p50:cRel) is activated in response to mitochondrial stress. Furthermore, we have shown that activation of the heterogeneous nuclear ribonuclear protein hnRNPA2, which acts as a transcriptional co-activator of nuclear oncogenes, occurs in response to mitochondrial stress and is critical for propagation of MtRS and tumorigenic reprogramming [21,36,63].

**Fig 5:**
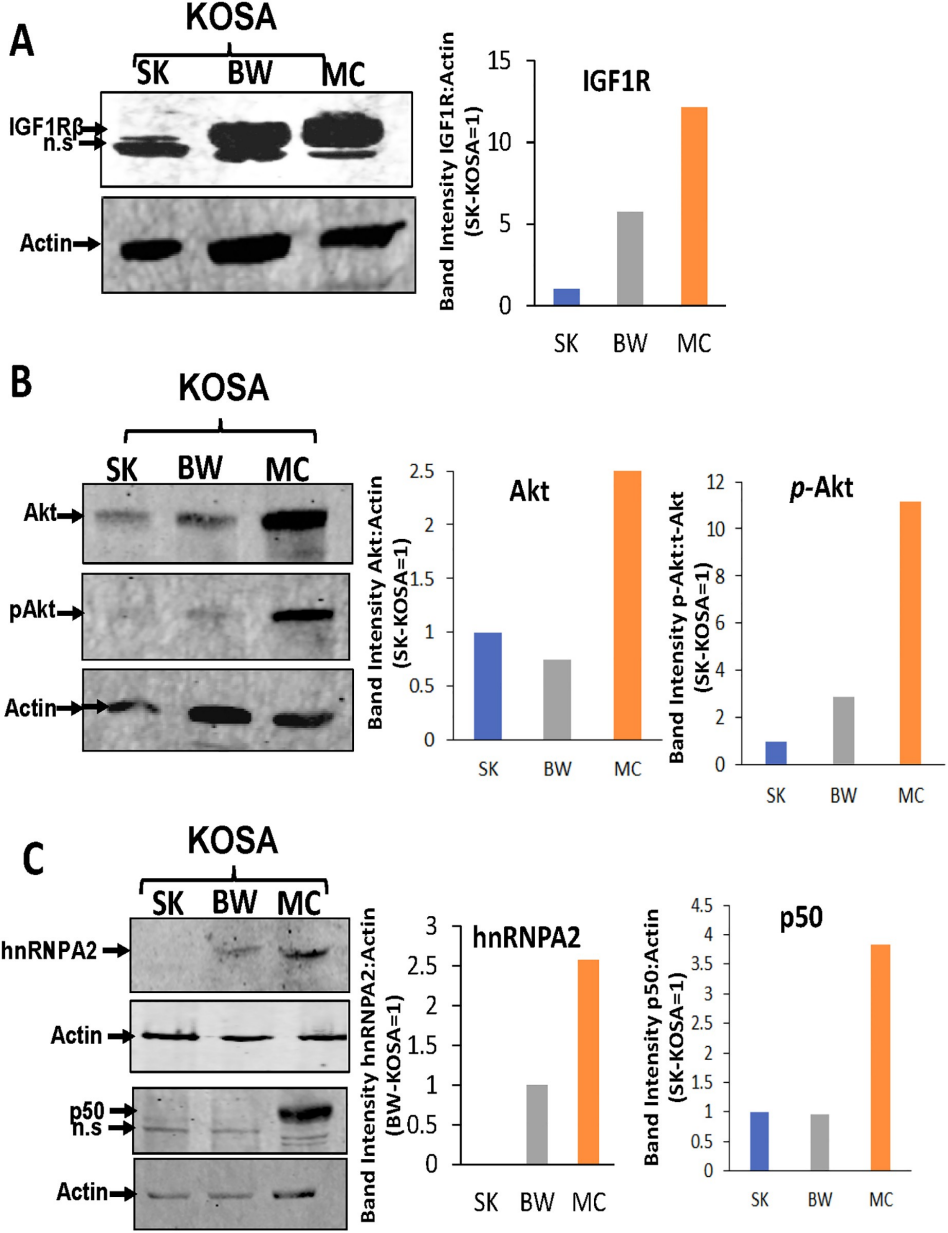
Induction of MtRS marker proteins in OSA cell lines. Western blot showing the expression levels of MtRS marker proteins: **(A)** IGF-1R, **(B)** Akt and **(C)** hnRNPA2 and p50 in total cell lysate. n.s = non-specific.

Based on these previous findings, we analyzed the protein expression levels of MtRS marker proteins in the OSA cell lines. We observed higher levels of the MtRS marker proteins IGF-1R, Akt1, hnRNPA2 and NFkB (p50) in MC-KOSA cells compared with the other cell lines, suggesting that MtRS induction correlates with greater mitochondrial dysfunction and genomic defects in this cell line (Fig 5A–5C). As mentioned in the materials and methods section, the antibodies used here have not been previously tested in canine species and therefore the faster migrating bands we observed in the IGF-1Rβ and p50 western blots are possibly non-specific immunoreactive proteins. The higher levels of MtRS protein expression suggests the activation of the signaling pathway in response to mitochondrial dysfunction is dependent on the extent of the depletion of mtDNA.

### Induction of OSA aggressive phenotype in response to mitochondrial stress

**Fig 6:**
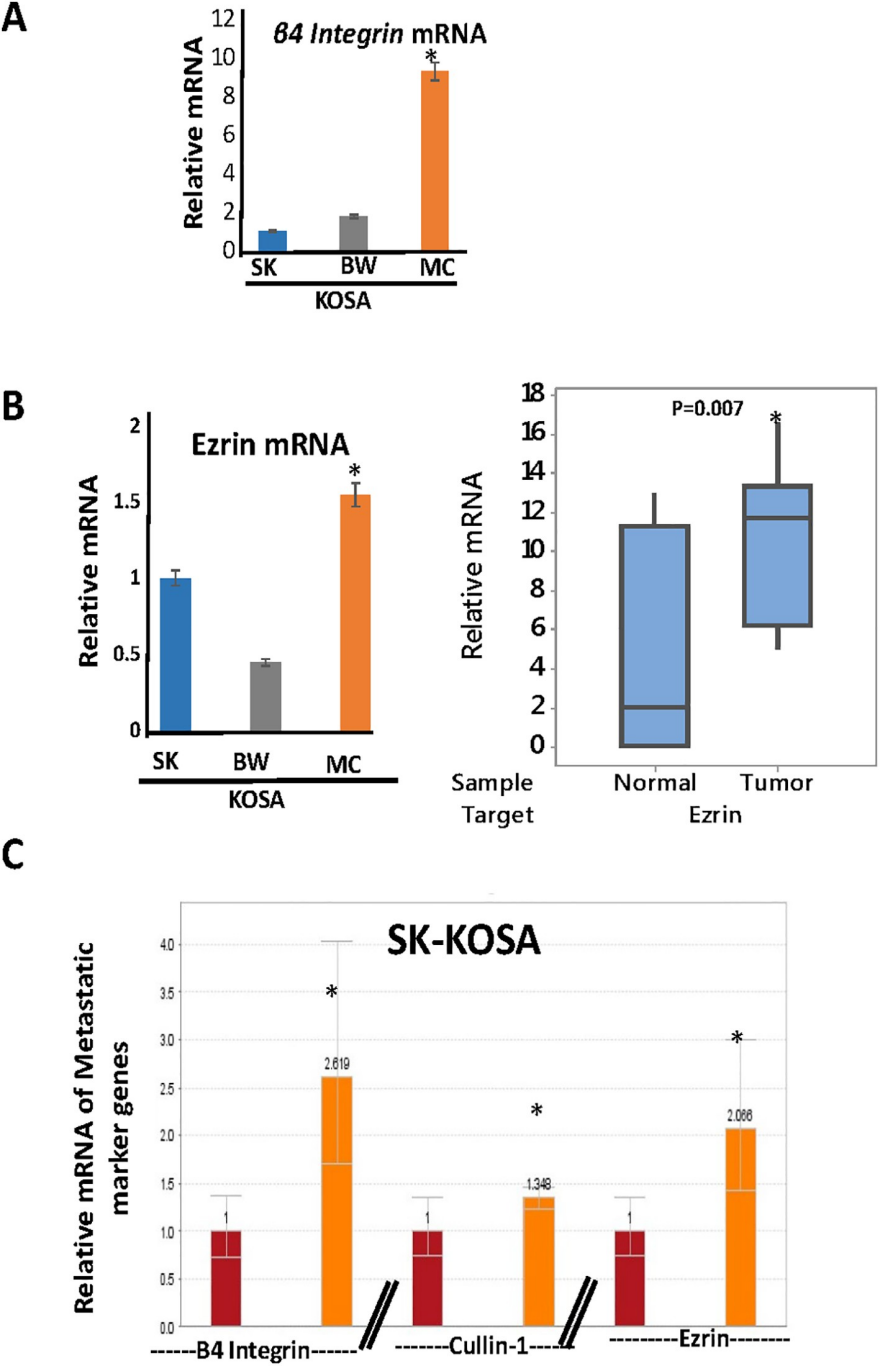
Prognostic marker expression in OSA cell lines. **(A)** Real time PCR of mRNA was used to quantify expression levels of the metastasis gene β4 Integrin in three OSA cell lines. **(B)** Real time PCR of mRNA was used to quantify expression levels of the metastasis gene Ezrin in three OSA cell lines (left) and canine non-tumor and tumor tissues (right). **(C)** Expression levels of the three marker genes of aggressive OSA, β4 Integrin, Cullin-1, and Ezrin were quantified using real time PCR of mRNA from SK-KOSA and mtDNA-depleted SK-KOSA.

Biomarkers to identify aggressive potential of primary osteosarcomas are not well defined. Some markers reported to be associated with aggressive osteosarcomas are β4 Integrin, Ezrin, and Cullin-1. Integrins, such as β4 Integrin, are heterodimers comprised of α and β subunits and regulate many biological processes such as cell adhesion, signaling, migration, proliferation, survival, angiogenesis, oncogenesis, and metastasis [64–68]. We observed that the mRNA expression level of β4 Integrin is higher in MC-KOSA cells compared to SK-KOSA and BW-KOSA, which correlates with the higher levels of mitochondrial dysfunction in these cells (Fig 6A).

Ezrin is a cytoskeleton linker protein that belongs to the ezrin, radixin, and moesin (ERM) family [69]. It is reported to influence the cellular processes involved in metastatic transition by physically connecting the actin cytoskeleton with the cell membrane and thus engaging the cancer microenvironment [70]. We observed that the mRNA level of Ezrin is higher in MC-KOSA cells compared to SK-KOSA and BW-KOSA. OSA tumors, which contained lower mtDNA copies (Fig 1A), have higher Ezrin mRNA compared to matched non-tumor control tissues which further suggests the correlation between mtDNA content and tumorigenesis (Fig 6B).

To address if reduction of mtDNA content played a causal role in induction of the marker genes of aggressive OSA, we selected SK-KOSA cells, which had the highest mtDNA content and low expression of the tumor marker genes, and investigated the effect of mtDNA depletion in this cell line. Compared to parental SK-KOSA cells, mtDNA depletion in SK-KOSA cells resulted in higher levels of the markers β4 Integrin, Cullin-1 and Ezrin suggesting a causal role of mtDNA depletion in the induction of the marker genes that drive tumor progression (Fig 6C).

**Fig 7:**
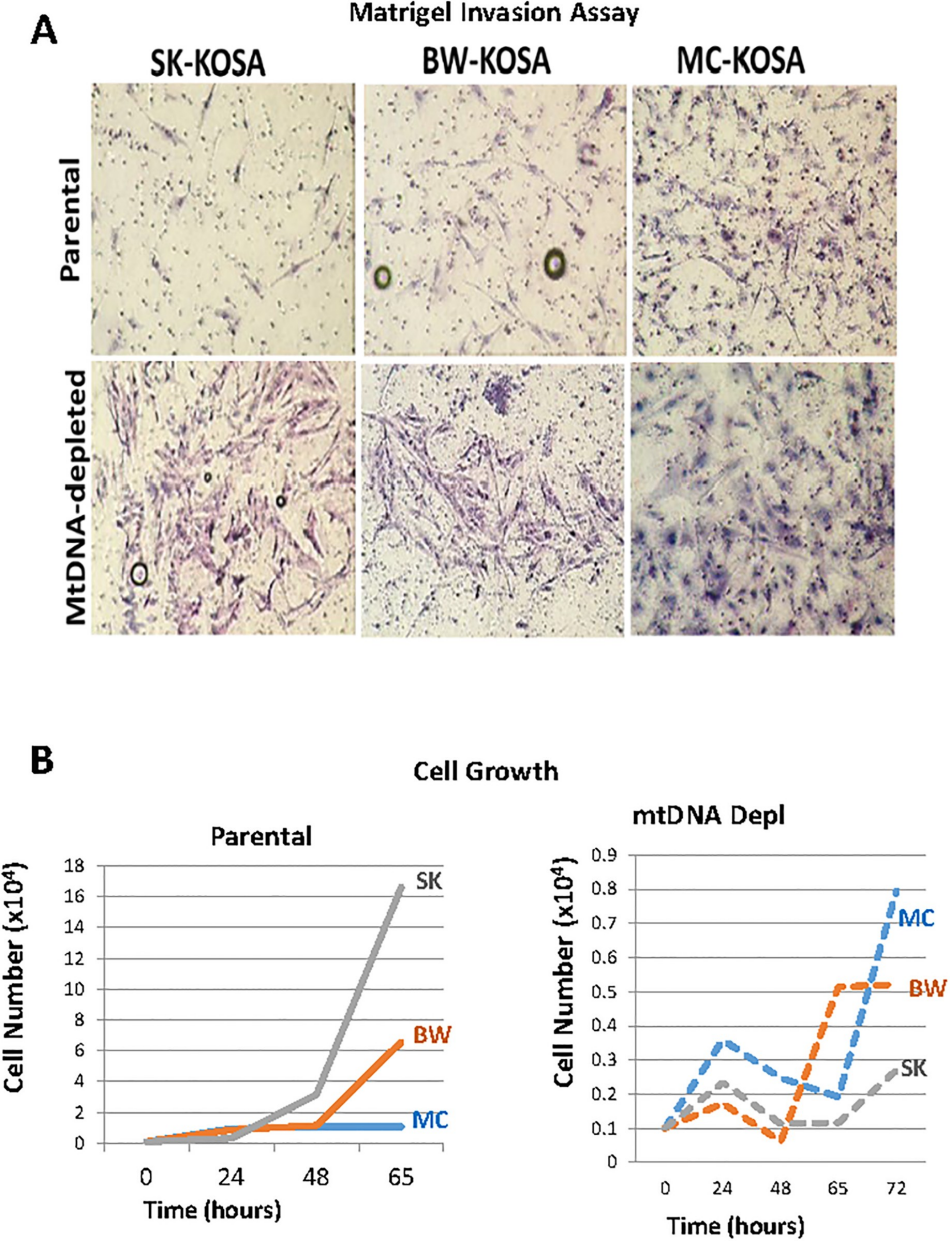
Acquired Invasive potential in mtDNA depleted cells. **(A)** Invasive potential of three parental OSA cell lines compared to three mtDNA-depleted OSA cell lines as observed in a Matrigel invasion assay. **(B)** Plot of the growth rate of three parental OSA and mtDNA-depleted OSA cell lines over the course of 72 hours.

The cellular processes involved in tumor progression towards metastasis is complex. During initial stages of the metastatic process, aggressive tumor cells from within the primary tumor acquire the potential to invade into the surrounding host tissue. This invasive potential is recapitulated in an *in vitro* Matrigel invasion assay where the capacity of cells to invade and migrate through the Matrigel layer enriched with components of the tumor microenvironment is assessed. We found that SK-KOSA and BW-KOSA are non-invasive, while MC-KOSA is highly invasive as shown by the cells that migrated across the Matrigel layer (Fig 7A, top panel) further suggesting the association of low mtDNA content with invasive phenotype. Interestingly, chemically depleting mtDNA content in the non-invasive SK-KOSA and BW–KOSA cells resulted in these cells acquiring an invasive phenotype (Fig 7A, lower panel) thereby suggesting a causal role of mtDNA depletion towards invasive potential of tumor cells. As expected, depletion of mtDNA content in the low mtDNA containing, highly invasive MC-KOSA cells, did not have any further effect on their invasive potential.

The differences in the invasive potential among the cell lines is independent of the cell proliferation as shown in Fig 7B (Bottom panel). MC-KOSA which has high invasiveness among the three cell lines, has the lowest cell proliferation rate (Fig 7B, left panel). The EtBr treated mtDNA-depleted SK-KOSA and BW-KOSA are highly invasive have lower cell growth rate compared to their parental cells (Fig 7B, right panel). While increased cell proliferation is typical during tumor initiation, higher cell proliferation is not a requirement for tumor progression (invasion, metastasis). Therefore, our result here is in agreement with reports that there is no correlation between cell proliferation and aggressive potential of the primary tumor.

Taken together, these data suggest a causal role between reduced mtDNA content and associated mitochondrial dysfunction and development of an invasive, metastatic phenotype.

## Discussion

Osteosarcomas in canine and human patients share common clinical, biological, behavioral and genetic features. The disease is highly metastatic in both species and effective therapies for the treatment or prevention of metastatic disease remain elusive. Although human and canine patients differ in the average age of onset of the disease [71–73] they share clinical and molecular prognostic markers. Mitochondrial defects have been associated with aggressive phenotypes in many different cancer types, and low mtDNA content has also been associated with chemoresistance and poor prognosis [28,39–41,74,75]. Our earlier reports, which show that mtDNA dysfunction-induced mitochondria-to-nucleus retrograde signaling (MtRS) pathway [21,31,32,34,35,37,62] influences tumorigenesis and our findings reported here suggest that mitochondrial defects and MtRS markers could potentially be biomarkers for identifying highly aggressive OSA.

Although the prognostic relevance of mitochondrial defects has been reported in many cancers, to date there has been very little information on the relationship between mitochondrial defects in canine OSA and their association with tumor aggression. In this study our goal was to determine whether mtDNA content and dysfunction were associated with an aggressive phenotype in canine OSA and whether we could identify mitochondrial dysfunction-associated molecular markers which in future can be used as therapeutic targets. We observed inherent heterogeneity in mtDNA content and mitochondrial dysfunction amongst the OSA cell lines and tumors which reflects the heterogeneity seen in OSA progression and prognosis in canine and human OSA patients. Furthermore, we identified mitochondrial genomic and functional defects in canine OSA cell lines and primary tumors that could potentially influence tumor progression and metastasis.

In addition to low mtDNA copy number, we identified marked loss of the mitochondrial filamentous network as well as altered mitochondrial fission. These correlated with the extent of reduction in mtDNA copy number and invasive potential, suggesting that reduction in mtDNA copy number could be used as a prognostic marker for OSA. The changes in mitochondrial fission in the most aggressive MC-KOSA cell line suggests that targeting mitochondrial fission may provide a therapeutic opportunity to rescue mitochondrial functions and reverse the metastatic progression. Notably, therapeutic targeting of mitochondria fission by mDivi-1, a DRP1 inhibitor, has been shown to abrogate mitochondrial stress and reverse the tumorigenic phenotype [76].

Our findings that impairment of mitochondrial electron transport chain function is dependent on mtDNA content further confirms the interdependence of mitochondrial membrane potential and an efficient respiration process reported earlier [77]. In agreement with these earlier studies, we now demonstrate that mtDNA depletion in canine OSA cell lines induces the activation of genes associated with MtRS including Akt1, IGF-1R and hnRNPA2. Importantly, our results show that mtDNA depletion induced *β4Integrin* and *Ezrin* genes in the non-invasive SK-KOSA cell line resulting in an acquired invasive phenotype, which suggests that mitochondrial dysfunction plays a critical role in the complex metastatic process.

This study lays the foundation for evaluating agents that rescue mitochondrial functions and/or block the aberrant MtRS pathway, inhibit glycolysis, or down regulate the expression of MtRS markers, which might provide new avenues of therapy for preventing metastatic disease in both human and canine OSA patients.

## Supporting information

S1 Fig Depletion of mtDNA copies in OSA cell lines. Parental OSA cell lines compared to OSA cell lines treated with 50 ng/mL EtBr for three passages. The mtDNA content (y-axis) is analyzed from total DNA as the copy number of mtDNA gene CcO1, normalized to the copy number of nuclear single copy gene CcOIVi1.
(TIFF)

S2 Fig Expression of ETC proteins in OSA cell lines in response to mtDNA depletion. Immunofluorescence images showing ATPB (red) and DAPI (nuclei in blue) or CcOIVi1 (green) and DAPI (nuclei in blue) in parental and mtDNA depleted **(A)** SK-KOSA, **(B)** BW-KOSA and **(C)** MC-KOSA cell lines as indicated. Scale bar: 10μm, magnification 100x.
(TIFF)

S3 Fig Expression of mitochondrial fusion marker protein OPA1. **(A)** OSA cell lines stained for mitochondrial fusion marker protein OPA1 and nuclei (DAPI in blue) viewed under a Leica widefield microscope. Scale bar: 10μm, magnification 100x. **(B)** Top panel: Western Immunoblot showing low OPA1 protein levels in MC-KOSA relative to SK-KOSA and BW-KOSA. Bottom Panel: Quantitation (densitometry) of the protein levels.
(TIFF)
