# Supplementary figures and images for: Mitochondrial genome and functional defects in osteosarcoma are associated with their aggressive phenotype
Source: PLoS One. 2018 Dec 21;13(12):e0209489. doi: 10.1371/journal.pone.0209489 (PMC6303035; doi:10.1371/journal.pone.0209489)

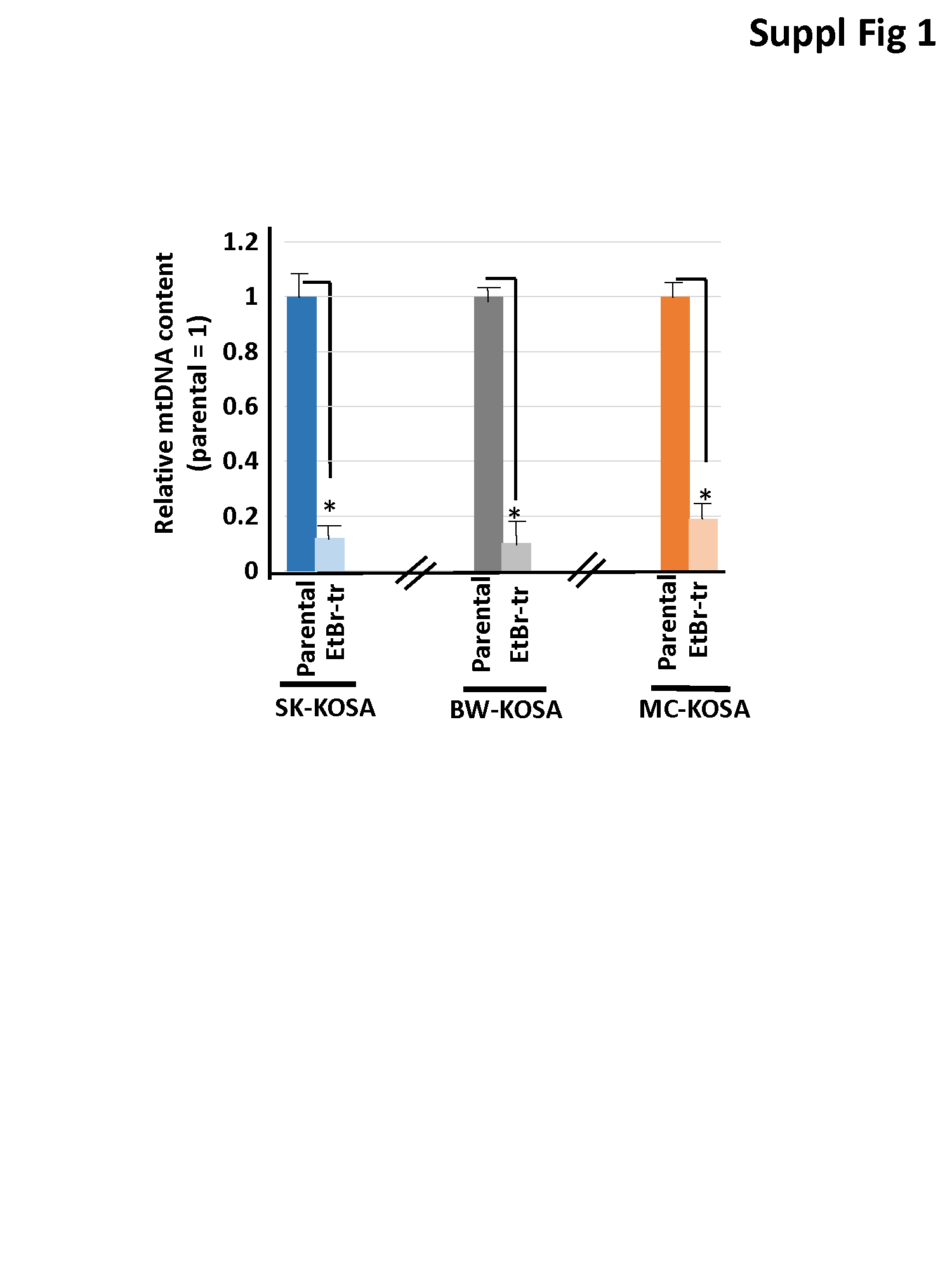

Supplement: S1 Fig — Parental OSA cell lines compared to OSA cell lines treated with 50 ng/mL EtBr for three passages. The mtDNA content (y-axis) is analyzed from total DNA as the copy number of mtDNA gene CcO1, normalized to the copy number of nuclear single copy gene CcOIVi1. (TIFF) [file pone.0209489.s001.tiff]

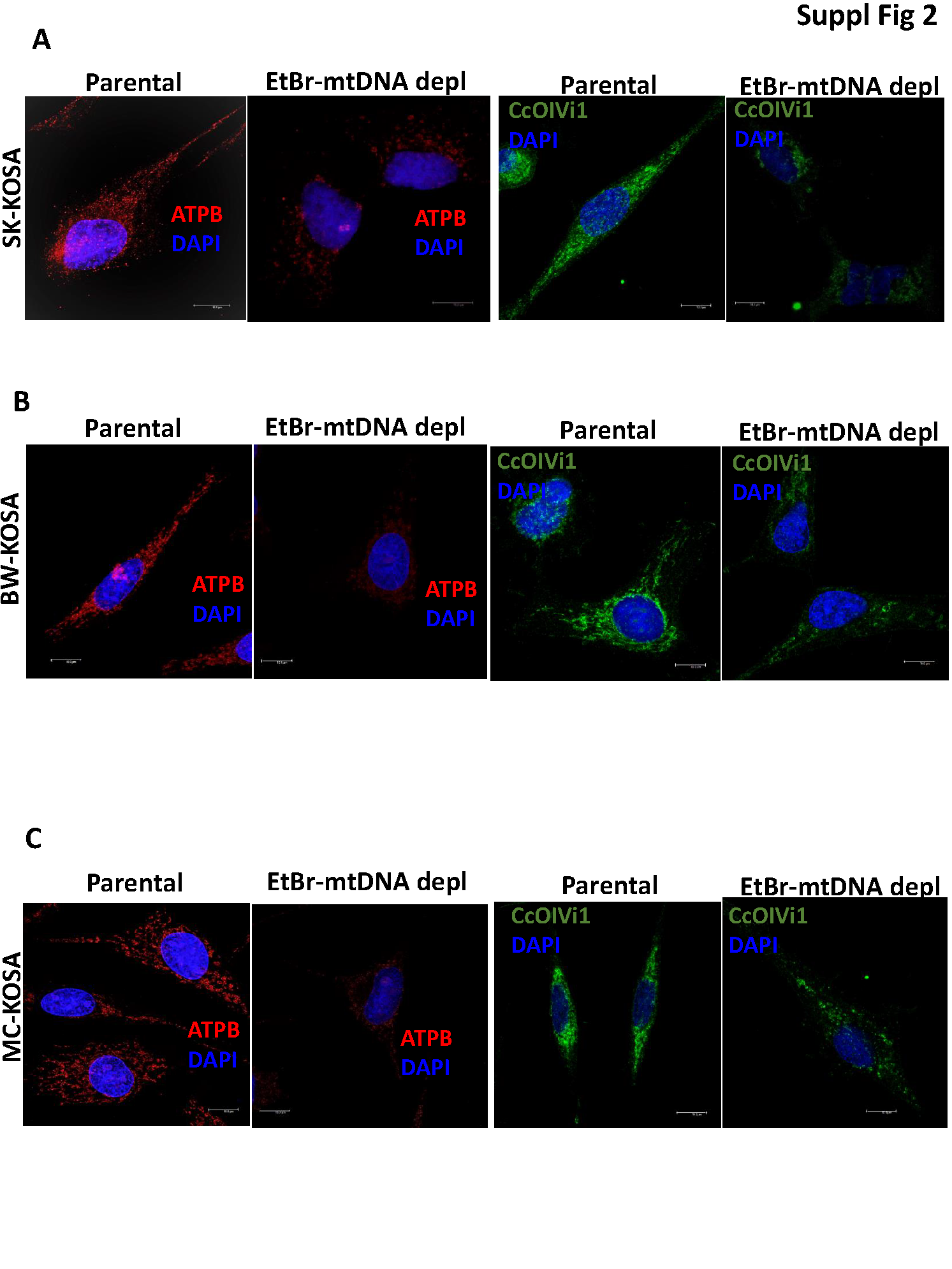

Supplement: S2 Fig — Immunofluorescence images showing ATPB (red) and DAPI (nuclei in blue) or CcOIVi1 (green) and DAPI (nuclei in blue) in parental and mtDNA depleted (A) SK-KOSA, (B) BW-KOSA and (C) MC-KOSA cell lines as indicated. Scale bar: 10μm, magnification 100x. (TIFF) [file pone.0209489.s002.tiff]

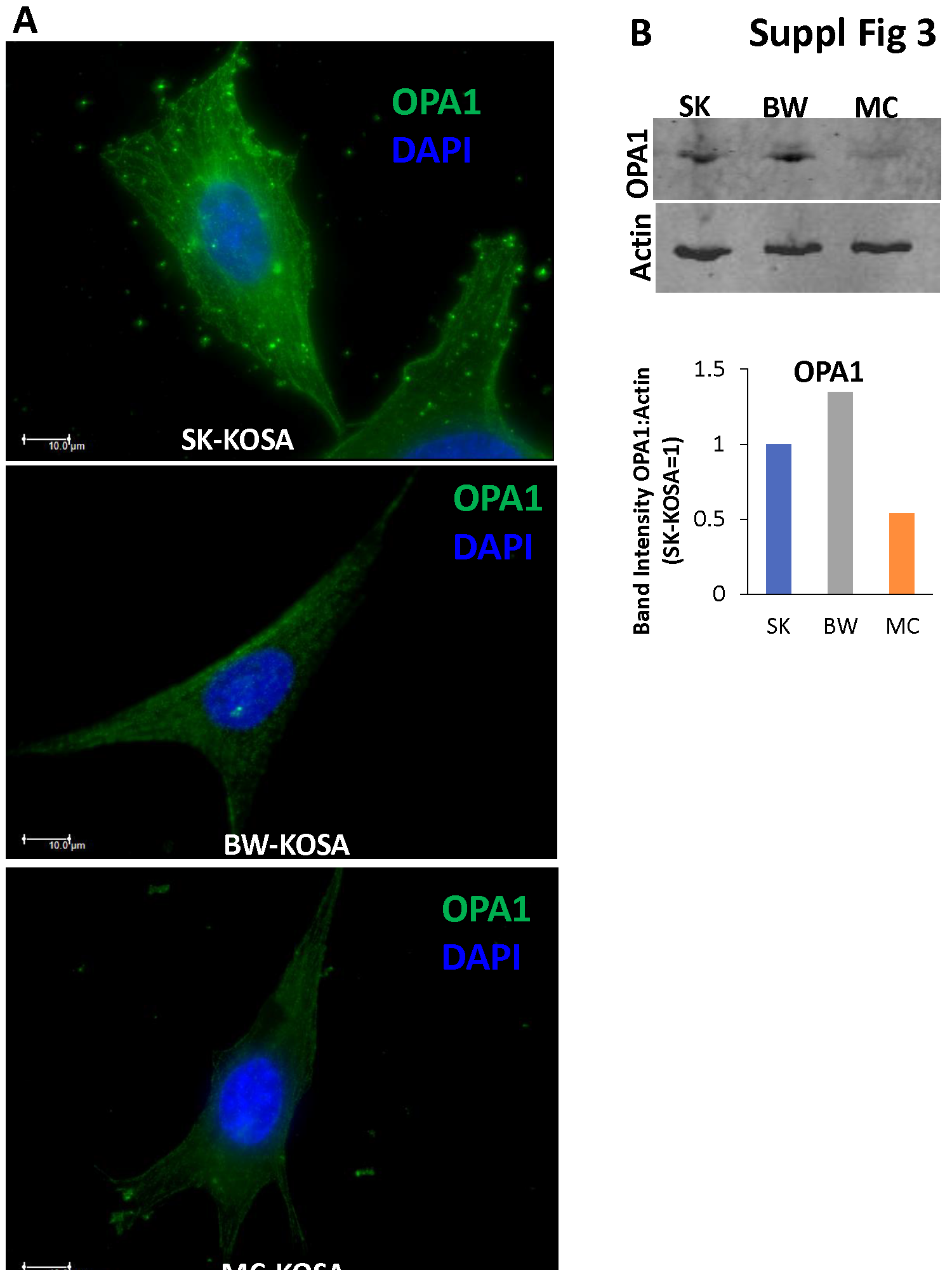

Supplement: S3 Fig — (A) OSA cell lines stained for mitochondrial fusion marker protein OPA1 and nuclei (DAPI in blue) viewed under a Leica widefield microscope. Scale bar: 10μm, magnification 100x. (B) Top panel: Western Immunoblot showing low OPA1 protein levels in MC-KOSA relative to SK-KOSA and BW-KOSA. Bottom Panel: Quantitation (densitometry) of the protein levels. (TIFF) [file pone.0209489.s003.tiff]
